# Supplementary material for: Associations of sunlight affinity with depression and sleep disorders in American males: Evidence from NHANES 2009–2020
Source: PLoS One. 2025 Oct 15;20(10):e0332098. doi: 10.1371/journal.pone.0332098 (PMC12527189; doi:10.1371/journal.pone.0332098)
Supplement: S1 Table — N = sample size. (DOCX) [file pone.0332098.s001.docx]

**S1 Table. **Pearson correlation matrix between sunlight exposure duration and sunlight preference scores.****

| **Variable** | **Sunlight exposure duration (hours)** | **Sunlight preference score (score)** |
| --- | --- | --- |
| Sunlight exposure duration (hours) |  | |
| Pearson’s r | 1 | 0.184 |
| p-value (two-tailed) | — | <0.001 |
| N | 7306 | 7306 |
| Sunlight preference score (scores) |  | |
| Pearson’s r | 0.184 | 1 |
| p-value (two-tailed) | <0.001 | — |
| N | 7306 | 7306 |

N = sample size.
