# Supplementary material for: Associations of sunlight affinity with depression and sleep disorders in American males: Evidence from NHANES 2009–2020
Source: PLoS One. 2025 Oct 15;20(10):e0332098. doi: 10.1371/journal.pone.0332098 (PMC12527189; doi:10.1371/journal.pone.0332098)
Supplement: S2 Table — CI: Confidence Interval. (DOCX) [file pone.0332098.s002.docx]

**S2 **Table. Univariate linear regression analysis of sunlight preference score and sunlight exposure duration.****

| **Independent Variable** | **Dependent Variable** | **β (95% CI)** | **Standard Error** | **t-value** | **p-value** |
| --- | --- | --- | --- | --- | --- |
| Sunlight preference score (scores) | Sunlight exposure duration (hours) | 0.35 (0.31–0.40) | 0.02 | 15.98 | <0.001 |
| Sunlight exposure duration (hours) | Sunlight preference score (scores) | 0.10 (0.08–0.11) | 0.01 | 15.98 | <0.001 |

CI: Confidence Interval
