# Supplementary material for: Associations of sunlight affinity with depression and sleep disorders in American males: Evidence from NHANES 2009–2020
Source: PLoS One. 2025 Oct 15;20(10):e0332098. doi: 10.1371/journal.pone.0332098 (PMC12527189; doi:10.1371/journal.pone.0332098)
Supplement: S3 Table — SPS, sunlight preference score; SED, sunlight exposure duration; StD, subthreshold depression; MDD, major depressive disorder; OR, odds ratio; aOR, adjusted odds ratio; CI, confidence interval. Model 1 was unadjusted. Model 2 was adjusted for demographics. (DOCX) [file pone.0332098.s003.docx]

**S3 Table. Logistic regression models for the association of sunlight affinity with depression and sleep disorders.**

| **Variables** | | **StD** | | | | **MDD** | | | |
| --- | --- | --- | --- | --- | --- | --- | --- | --- | --- |
|  |  | **Model 1** | | **Model 2** | | **Model 1** | | **Model 2** | |
|  |  | **OR (95% CI)** | **P** | **aOR (95% CI)** | **P** | **OR (95% CI)** | **P** | **aOR (95% CI)** | **P** |
| **SPS** | Scores | 0.89 (0.82–0.98) | 0.017 | 0.87 (0.79–0.95) | 0.003 | 0.82 (0.71–0.94) | 0.005 | 0.78 (0.68–0.90) | <0.001 |
|  | Categories |  | | | | | | | |
|  | Negative attitude | Reference | | Reference | | Reference | | Reference | |
|  | Neutral attitude | 0.92 (0.72–1.18) | 0.513 | 0.89 (0.69–1.15) | 0.375 | 0.64 (0.44–0.94) | 0.025 | 0.60 (0.40–0.90) | 0.015 |
|  | Positive attitude | 0.73 (0.55–0.95) | 0.024 | 0.67 (0.50–0.88) | 0.006 | 0.57 (0.38–0.84) | 0.006 | 0.48 (0.31–0.73) | 0.001 |
|  | P-trend | 0.85 (0.75–0.97) | 0.020 | 0.81 (0.71–0.94) | 0.005 | 0.75 (0.61–0.92) | 0.008 | 0.69 (0.55–0.86) | 0.002 |
| **SED** | Hours | 0.99 (0.94–1.04) | 0.650 | 0.96 (0.91–1.01) | 0.098 | 0.95 (0.88–1.01) | 0.126 | 0.90 (0.84–0.96) | 0.004 |
|  | Categories |  | | | | | | | |
|  | Q1 | Reference | | Reference | | Reference | | Reference | |
|  | Q2 | 0.66 (0.48–0.89) | 0.008 | 0.69 (0.51–0.93) | 0.019 | 0.90 (0.56–1.44) | 0.651 | 0.99 (0.61–1.60) | 0.962 |
|  | Q3 | 0.70 (0.52–0.93) | 0.016 | 0.69 (0.52–0.93) | 0.016 | 0.89 (0.59–1.32) | 0.555 | 0.89 (0.60–1.33) | 0.568 |
|  | Q4 | 0.78 (0.58–1.03) | 0.083 | 0.68 (0.52–0.91) | 0.009 | 0.81 (0.57–1.15) | 0.240 | 0.65 (0.46–0.93) | 0.022 |
|  | P-trend | 0.98 (0.92–1.04) | 0.522 | 0.95 (0.89–1.00) | 0.064 | 0.96 (0.88–1.05) | 0.359 | 0.90 (0.83–0.99) | 0.028 |
| **Variables** | | **Short sleep** | | | | **Trouble sleeping** | | | |
|  |  | **Model 1** | | **Model 2** | | **Model 1** | | **Model 2** | |
|  |  | **OR (95% CI)** | **P** | **aOR (95% CI)** | **P** | **OR (95% CI)** | **P** | **aOR (95% CI)** | **P** |
| **SPS** | Scores | 1.09 (1.03–1.16) | 0.005 | 1.11 (1.04–1.18) | 0.002 | 0.92 (0.84–1.00) | 0.050 | 0.89 (0.82–0.98) | 0.016 |
|  | Categories |  | | | | | | | |
|  | Negative attitude | Reference | | Reference | | Reference | | Reference | |
|  | Neutral attitude | 0.93 (0.78–1.12) | 0.460 | 0.99 (0.83–1.19) | 0.944 | 0.94 (0.77–1.15) | 0.557 | 0.86 (0.70–1.06) | 0.157 |
|  | Positive attitude | 1.26 (1.06–1.49) | 0.010 | 1.32 (1.11–1.58) | 0.003 | 0.84 (0.64–1.09) | 0.196 | 0.78 (0.60–1.02) | 0.073 |
|  | P-trend | 1.14 (1.04–1.24) | 0.004 | 1.16 (1.06–1.27) | 0.001 | 0.91 (0.80–1.04) | 0.189 | 0.89 (0.77–1.01) | 0.081 |
| **SED** | Hours | 1.06 (1.01–1.10) | 0.011 | 1.05 (1.01–1.09) | 0.033 | 0.93 (0.90–0.97) | <0.001 | 0.94 (0.90–0.97) | 0.002 |
|  | Categories |  | | | | | | | |
|  | Q1 | Reference | | Reference | | Reference | | Reference | |
|  | Q2 | 0.78 (0.63–0.95) | 0.018 | 0.79 (0.64–0.98) | 0.034 | 1.10 (0.85–1.43) | 0.457 | 1.07 (0.83–1.40) | 0.590 |
|  | Q3 | 0.95 (0.80–1.13) | 0.578 | 0.96 (0.80–1.15) | 0.663 | 1.07 (0.87–1.31) | 0.527 | 1.00 (0.81–1.23) | 0.974 |
|  | Q4 | 1.11 (0.90–1.37) | 0.322 | 1.08 (0.87–1.34) | 0.483 | 0.77 (0.61–0.96) | 0.022 | 0.76 (0.60–0.96) | 0.022 |
|  | P-trend | 1.05 (1.01–1.10) | 0.026 | 1.04 (1.00–1.09) | 0.073 | 0.93 (0.89–0.97) | 0.002 | 0.93 (0.89–0.98) | 0.004 |

SPS, sunlight preference score; SED, sunlight exposure duration; StD, subthreshold depression; MDD, major depressive disorder; OR, odds ratio; aOR, adjusted odds ratio; CI, confidence interval.

Model 1 was unadjusted.

Model 2 was adjusted for age, PIR, race, education and marital status.
