# Supplementary material for: Associations of sunlight affinity with depression and sleep disorders in American males: Evidence from NHANES 2009–2020
Source: PLoS One. 2025 Oct 15;20(10):e0332098. doi: 10.1371/journal.pone.0332098 (PMC12527189; doi:10.1371/journal.pone.0332098)
Supplement: S4 Table — SPS, sunlight preference score; SED, sunlight exposure duration; StD, subthreshold depression; MDD, major depressive disorder; AIC, Akaike information criterion; BIC, Bayesian information criterion. Model 1 was without adjustment for covariates. Model 2 adjusted for demographic covariates (including age, race, education, marital status and PIR). Model 3 further adjusted for lifestyle (including smoking history, alcohol consumption history, physical activity, and BMI) and comorbidities (including CVD, liver condition, asthma, weak/failing kidneys, cancer/malignancy, diabetes, and hypertension) based on Model 2. (DOCX) [file pone.0332098.s004.docx]

**S4 Table. Goodness-of-fit statistics for the logistic regression models.**

| **Variables** | **Model** | **StD** | | **MDD** | | **Short sleep** | | **Trouble sleeping** | |
| --- | --- | --- | --- | --- | --- | --- | --- | --- | --- |
|  |  | **AIC** | **BIC** | **AIC** | **BIC** | **AIC** | **BIC** | **AIC** | **BIC** |
| **SPS (scores)** | Model 1 | 5737 | 5763 | 2917 | 2931 | 9628 | 9655 | 7375 | 7389 |
|  | Model 2 | 5626 | 5750 | 2742 | 2830 | 9524 | 9641 | 7104 | 7193 |
|  | Model 3 | 5592 | 5715 | 2655 | 2823 | 9482 | 9614 | 6825 | 6997 |
| **SED (hours)** | Model 1 | 5748 | 5773 | 2918 | 2931 | 9626 | 9660 | 7373 | 7387 |
|  | Model 2 | 5639 | 5762 | 2733 | 2821 | 9528 | 9640 | 7108 | 7197 |
|  | Model 3 | 5602 | 5728 | 2645 | 2814 | 9488 | 9618 | 6824 | 6997 |

SPS, sunlight preference score; SED, sunlight exposure duration; StD, subthreshold depression; MDD, major depressive disorder; AIC, Akaike information criterion; BIC, Bayesian information criterion.

Model 1 was without adjustment for covariates. Model 2 adjusted for demographic covariates (including age, race, education, marital status and PIR). Model 3 further adjusted for lifestyle (including smoking history, alcohol consumption history, physical activity, and BMI) and comorbidities (including CVD, liver condition, asthma, weak/failing kidneys, cancer/malignancy, diabetes, and hypertension) based on Model 2.
