# Supplementary material for: Associations of sunlight affinity with depression and sleep disorders in American males: Evidence from NHANES 2009–2020
Source: PLoS One. 2025 Oct 15;20(10):e0332098. doi: 10.1371/journal.pone.0332098 (PMC12527189; doi:10.1371/journal.pone.0332098)
Supplement: S5 Table — SPS, sunlight preference score; SED, sunlight exposure duration; StD, subthreshold depression; MDD, major depressive disorder; CI, confidence interval. Adjusted for demographics, lifestyle, and comorbidities. (DOCX) [file pone.0332098.s005.docx]

**S5 Table. Mediation analyses results.**

| **Exposure → Mediator → Outcome** | **Effect Type** | **β (95% CI)** | **P** | **Prop. Mediated (95% CI)** |
| --- | --- | --- | --- | --- |
| Sleep hours → SPS → PHQ-9 scores | Indirect effect | 0.0126 (0.0043, 0.0200) | <0.001 | -0.300 (-0.389, -0.030) |
|  | Direct effect | -0.0544 (-0.2250, -0.0300) | 0.004 |  |
|  | Total Effect | -0.0419 (-0.2150, -0.0300) | 0.014 |  |
| PHQ-9 scores → SPS → Sleep hours | Indirect effect | 0.0015 (0.0006, 0.0020) | <0.001 | -0.290 (-0.390, -0.020) |
|  | Direct effect | -0.0068 (-0.0320, 0.0000) | 0.004 |  |
|  | Total Effect | -0.0053 (-0.0300, 0.0000) | 0.014 |  |
| Trouble sleeping → SPS → PHQ-9 scores | Indirect effect | 0.0069 (0.0015, 0.0300) | 0.020 | 0.003 (0.001, 0.010) |
|  | Direct effect | 2.4917 (2.2310, 2.8000) | <0.001 |  |
|  | Total Effect | 2.4986 (2.2490, 2.8200) | <0.001 |  |
| PHQ-9 scores → SPS → Trouble sleeping | Indirect effect | 0.0000 (-0.0001, 0.0000) | 0.240 | 0.001 (-0.002, 0.010) |
|  | Direct effect | 0.0279 (0.0230, 0.0300) | <0.001 |  |
|  | Total Effect | 0.0279 (0.0230, 0.0300) | <0.001 |  |
| StD → SPS → Short Sleep | Indirect effect | -0.0026 (-0.0060, 0.0000) | <0.001 | -0.039 (-0.073, -0.010) |
|  | Direct effect | 0.0698 (0.0660, 0.1400) | <0.001 |  |
|  | Total Effect | 0.0672 (0.0620, 0.1400) | <0.001 |  |
| Short Sleep → SPS → StD | Indirect effect | -0.0016 (-0.0032, 0.0000) | <0.001 | -0.042 (-0.073, -0.010) |
|  | Direct effect | 0.0387 (0.0350, 0.0800) | <0.001 |  |
|  | Total Effect | 0.0371 (0.0330, 0.0800) | <0.001 |  |
| MDD → SPS → Trouble sleeping | Indirect effect | 0.0019 (-0.0006, 0.0019) | 0.130 | 0.004 (-0.001, 0.020) |
|  | Direct effect | 0.5228 (0.3770, 0.5000) | <0.001 |  |
|  | Total Effect | 0.5247 (0.3800, 0.5000) | <0.001 |  |
| Trouble sleeping → SPS → MDD | Indirect effect | 0.0020 (0.0001, 0.0020) | 0.022 | 0.011 (0.001, 0.020) |
|  | Direct effect | 0.1769 (0.1210, 0.1800) | <0.001 |  |
|  | Total Effect | 0.1789 (0.1220, 0.1800) | <0.001 |  |
| PHQ-9 scores → SED → Sleep hours | Indirect effect | 0.0016 (0.0004, 0.0020) | <0.001 | -0.305 (-0.303, -0.020) |
|  | Direct effect | -0.0069 (-0.0320, 0.0000) | 0.004 |  |
|  | Total Effect | -0.0053 (-0.0300, 0.0000) | 0.014 |  |
| Sleep hours → SED → PHQ-9 scores | Indirect effect | 0.0129 (0.0025, 0.0100) | <0.001 | -0.309 (-0.300, -0.010) |
|  | Direct effect | -0.0548 (-0.2220, -0.0300) | 0.004 |  |
|  | Total Effect | -0.0419 (-0.2150, -0.0300) | 0.014 |  |
| PHQ-9 scores → SED → Trouble sleeping | Indirect effect | 0.0003 (-0.0000, 0.0000) | 0.060 | 0.010 (-0.000, 0.010) |
|  | Direct effect | 0.0276 (0.0230, 0.0300) | <0.001 |  |
|  | Total Effect | 0.0279 (0.0230, 0.0300) | <0.001 |  |
| Trouble sleeping → SED → PHQ-9 scores | Indirect effect | 0.0194 (0.0059, 0.0300) | 0.002 | 0.008 (0.002, 0.010) |
|  | Direct effect | 2.4793 (2.2320, 2.8000) | <0.001 |  |
|  | Total Effect | 2.4986 (2.2490, 2.8200) | <0.001 |  |
| Short Sleep → SED → Trouble sleeping | Indirect effect | -0.0045 (-0.0050, 0.0000) | 0.002 | -0.132 (-0.074, -0.010) |
|  | Direct effect | 0.0386 (0.0350, 0.0900) | <0.001 |  |
|  | Total Effect | 0.0341 (0.0330, 0.0800) | <0.001 |  |
| Trouble sleeping → SED → Short Sleep | Indirect effect | -0.0055 (-0.0050, 0.0000) | 0.002 | -0.129 (-0.071, -0.010) |
|  | Direct effect | 0.0482 (0.0440, 0.1100) | <0.001 |  |
|  | Total Effect | 0.0427 (0.0410, 0.1100) | <0.001 |  |

SPS, sunlight preference score; SED, sunlight exposure duration; StD, subthreshold depression; MDD, major depressive disorder; CI, confidence interval.

Adjusted for demographics, lifestyle, and comorbidities.
