# Supplementary material for: Associations of sunlight affinity with depression and sleep disorders in American males: Evidence from NHANES 2009–2020
Source: PLoS One. 2025 Oct 15;20(10):e0332098. doi: 10.1371/journal.pone.0332098 (PMC12527189; doi:10.1371/journal.pone.0332098)
Supplement: S6 Table — SPS, sunlight preference score; SED, sunlight exposure duration; StD, subthreshold depression; MDD, major depressive disorder; OR, odds ratio; aOR, adjusted odds ratio; CI, confidence interval. Adjusted for demographics, lifestyle, and comorbidities. (DOCX) [file pone.0332098.s006.docx]

**S6 Table. Logistic regression results after exclusion of extreme values.**

| **Variables** | | **StD** | **P** | **MDD** | **P** | **Short sleep** | **P** | **Trouble sleeping** | **P** |
| --- | --- | --- | --- | --- | --- | --- | --- | --- | --- |
|  |  | **aOR (95% CI)** |  | **aOR (95% CI)** |  | **aOR (95% CI)** |  | **aOR (95% CI)** |  |
| **SPS** | Scores | 0.85 (0.76–0.94) | 0.003 | 0.78 (0.66–0.91) | 0.002 | 1.13 (1.06–1.20) | <0.001 | 0.92 (0.83–1.01) | 0.099 |
|  | Categories |  | | | | | | | |
|  | Negative attitude | Reference | | Reference | | Reference | | Reference | |
|  | Neutral attitude | 0.89 (0.68–1.15) | 0.370 | 0.60 (0.40–0.92) | 0.021 | 0.99 (0.83–1.19) | 0.953 | 0.91 (0.73–1.13) | 0.382 |
|  | Positive attitude | 0.67 (0.50–0.89) | 0.008 | 0.51 (0.33–0.77) | 0.003 | 1.35 (1.12–1.61) | 0.002 | 0.86 (0.65–1.14) | 0.300 |
|  | P-trend | 0.81 (0.71–0.94) | 0.007 | 0.71 (0.57–0.89) | 0.004 | 1.18 (1.07–1.29) | <0.001 | 0.93 (0.81–1.07) | 0.305 |
| **SED** | Hours | 0.97 (0.91–1.03) | 0.291 | 0.90 (0.83–0.98) | 0.013 | 1.06 (1.02–1.11) | 0.009 | 0.93 (0.89–0.98) | 0.004 |
|  | Categories |  | | | | | | | |
|  | Q1 | Reference | | Reference | | Reference | | Reference | |
|  | Q2 | 0.83 (0.64–1.07) | 0.161 | 1.17 (0.86–1.59) | 0.316 | 0.92 (0.76–1.12) | 0.406 | 1.15 (0.92–1.43) | 0.235 |
|  | Q3 | 0.87 (0.60–1.25) | 0.453 | 0.69 (0.46–1.05) | 0.089 | 1.15 (0.94–1.42) | 0.176 | 0.90 (0.73–1.10) | 0.308 |
|  | Q4 | 0.83 (0.62–1.12) | 0.222 | 0.69 (0.47–1.00) | 0.057 | 1.22 (0.99–1.51) | 0.064 | 0.76 (0.60–0.97) | 0.030 |
|  | P-trend | 0.97 (0.91–1.03) | 0.341 | 0.89 (0.81–0.98) | 0.018 | 1.06 (1.01–1.12) | 0.024 | 0.92 (0.88–0.97) | 0.004 |

SPS, sunlight preference score; SED, sunlight exposure duration; StD, subthreshold depression; MDD, major depressive disorder; OR, odds ratio; aOR, adjusted odds ratio; CI, confidence interval.

Adjusted for demographics, lifestyle, and comorbidities.
