# Supplementary material for: Associations of sunlight affinity with depression and sleep disorders in American males: Evidence from NHANES 2009–2020
Source: PLoS One. 2025 Oct 15;20(10):e0332098. doi: 10.1371/journal.pone.0332098 (PMC12527189; doi:10.1371/journal.pone.0332098)
Supplement: S7 Table — SPS, sunlight preference score; SED, sunlight exposure duration; StD, subthreshold depression; MDD, major depressive disorder; OR, odds ratio; aOR, adjusted odds ratio; CI, confidence interval. Adjusted for demographics, lifestyle, and comorbidities. (DOCX) [file pone.0332098.s007.docx]

**S7 Table. Threshold effect analysis results after exclusion of extreme values.**

| **Outcome** | **SPS–Short sleep** | | **SPS–Trouble sleeping** | |
| --- | --- | --- | --- | --- |
|  | **aOR (95% CI)** | **P** | **aOR (95% CI)** | **P** |
| Fitting model by standard linear regression | 1.08 (1.03–1.13) | 0.003 | 0.92 (0.86–0.98) | 0.007 |
| Fitting model by two-piecewise linear regression |  | | | |
| Inflection point | 3 | | 4 | |
| <Inflection point | 1.02 (0.79–1.30) | 0.902 | 0.92 (0.82–1.03) | 0.145 |
| ≥Inflection point | 1.17 (1.08–1.27) | <0.001 | 0.62 (0.48–0.81) | <0.001 |
| P for likelihood ratio test |  | 0.048 |  | 0.037 |

SPS, sunlight preference score; SED, sunlight exposure duration; StD, subthreshold depression; MDD, major depressive disorder; aOR, adjusted odds ratio; CI, confidence interval.

Adjusted for demographics, lifestyle, and comorbidities.
