# Supplementary figures and images for: Associations of sunlight affinity with depression and sleep disorders in American males: Evidence from NHANES 2009–2020
Source: PLoS One. 2025 Oct 15;20(10):e0332098. doi: 10.1371/journal.pone.0332098 (PMC12527189; doi:10.1371/journal.pone.0332098)

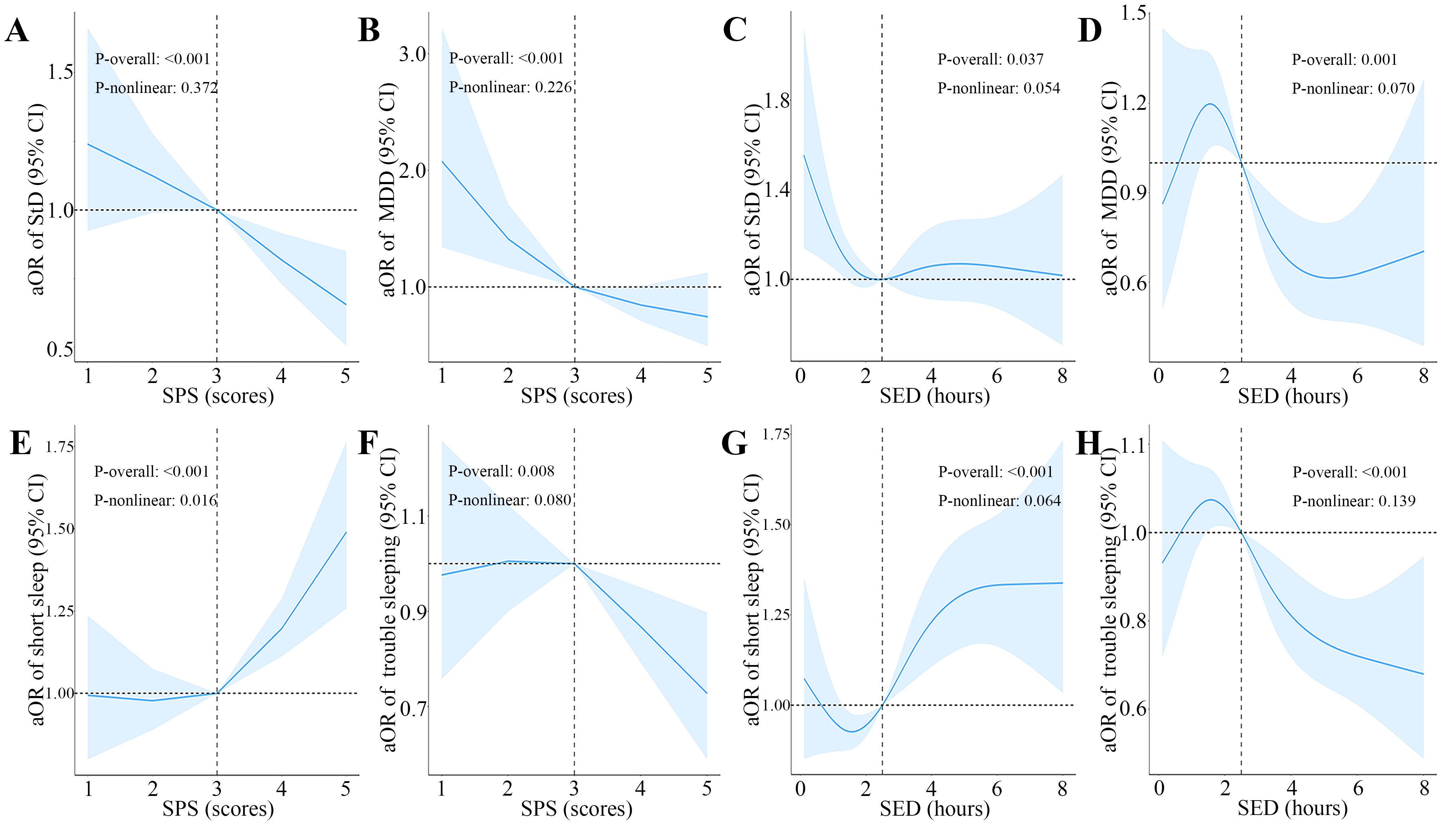

Supplement: S1 Fig — SPS, sunlight preference score; SED, sunlight exposure duration; StD, subthreshold depression; MDD, major depressive disorder; aOR, adjusted odds ratio; CI, confidence interval. (A) SPS is associated with StD, but no significant nonlinear relationship is observed. (B) SPS is associated with MDD, but no significant nonlinear relationship is observed. (C) SED is associated with StD, but no significant nonlinear relationship is observed. (D) SED is associated with MDD, but no significant nonlinear relationship is observed. (E) SPS shows a significant nonlinear association with short sleep. (F) SPS is associated with trouble sleeping, but no significant nonlinear relationship is observed. (G) SED is associated with short sleep, but no significant nonlinear relationship is observed. (H) SED is associated with trouble sleeping, but no significant nonlinear relationship is observed. Adjusted for demographics, lifestyle, and comorbidities. (TIF) [file pone.0332098.s008.tif]
