# Supplementary material for: Associations of sunlight affinity with depression and sleep disorders in American males: Evidence from NHANES 2009–2020
Source: PLoS One. 2025 Oct 15;20(10):e0332098. doi: 10.1371/journal.pone.0332098 (PMC12527189; doi:10.1371/journal.pone.0332098)
Supplement: Attachment — Submitted filename: Response to Reviewers.docx [file pone.0332098.s010.docx]

# Response Letter

Dear PLOS ONE Editorial Team,

We sincerely appreciate the opportunity to submit this revised manuscript entitled ”Associations of sunlight affinity with depression and sleep disorders in American males: Evidence from NHANES 2009-2020” (PONE-D-25-14823) to ***PLOS ONE***. We extend our deepest gratitude to the editorial team and reviewers for their substantial time investment and insightful critiques, which have significantly strengthened our work.

All reviewer comments have been meticulously addressed through comprehensive revisions. Below, we present the reviewers' comments in italics, followed by our point-by-point responses in **blue** text (with key modifications **highlighted in red**). Supporting documentation appears in standard font, while manuscript changes are tracked using **yellow highlighting**.

The detailed responses follow this section:

**Responses to Comments of Reviewer #1:**

***Comment 1: Was there data about the seasons/ sunlight per day in that location the subjects were selected from?***

Response:

We thank the reviewer for raising this crucial point regarding the potential influence of geographical and seasonal variations in sunlight availability. We acknowledge that this is an important limitation of our study. However, the NHANES database does not provide detailed meteorological data (e.g., daily sunlight hours, UV index) for each participant's location or the specific season of their interview. This limitation is inherent to the dataset and is a common challenge in large-scale national surveys.

To address this potential confounding factor as much as possible within the constraints of the available data, we took the following steps:

- Our assessment of Sunlight Exposure Duration (SED) was based on participants' self-reported average time spent outdoors over the previous 30 days. This period-based measure helps to mitigate the impact of day-to-day weather fluctuations and captures a more habitual behavior pattern.
- The consistency of our main findings in the sensitivity analyses, even after excluding participants with extreme values, suggests that the observed associations are robust and not solely driven by unmeasured seasonal outliers.

We recognize that the lack of granular environmental data may have influenced our results. We have explicitly acknowledged this limitation in the **Discussion** section of our manuscript and geo-coded meteorological data are warranted to confirm our findings and elucidate the precise role of seasonal and geographical sunlight variations.

Revised Manuscript with Track Changes (lines 439-442):

“Second, despite adjusting for multiple covariates, residual confounding from unmeasured environmental factors (e.g., detailed geographic location, seasonal and daily variations in sunlight intensity and duration, air pollution) may have influenced the results. The lack of this data is a limitation, and their inclusion in future research could help clarify the observed associations.”

***Comment 2: What is your conclusion about the fact that short sleep is related to sun exposure?***

Response:

We appreciate the reviewer for this insightful question regarding the positive association between sunlight exposure duration (SED) and short sleep, which indeed appears counterintuitive at first glance. Our conclusion, based on the totality of our findings, is that this relationship likely reflects a combination of behavioral patterns and physiological effects, and it may not be entirely negative. We interpret this association within a framework that distinguishes between **sleep quantity** (duration) and **sleep quality** (e.g., trouble sleeping). Our key observations are:

- Behavioral mechanism: Individuals with longer SED likely engage in more outdoor activities (e.g., work, exercise, socializing), which may compress available time for sleep, leading to a reduction in total sleep hours. This represents a socio-behavioral link where lifestyle choices associated with high sun exposure could incidentally limit sleep opportunity.
- **Physiological mechanism and a more nuanced view:** Crucially, our data suggest that while increased sun exposure is associated with shorter sleep duration, it is simultaneously associated with better sleep quality. This is strongly evidenced by the negative association we found between SED and trouble sleeping (OR = 0.94, 95% CI: 0.90–0.98). This indicates that although individuals with high sunlight affinity may sleep for a shorter period, they are less likely to suffer from sleep disturbances and insomnia symptoms. Furthermore, the non-linear (U-shaped) relationship we identified between SPS (sunlight preference score)/short sleep and SED/short sleep suggests the relationship is complex and not simply linear. The positive association manifests after a certain threshold, which may represent a point where the circadian-phase-shifting effects of light become significant.
- **Integration of dimensions:** The positive correlation between SPS (psychological preference) and SED (behavioral exposure) suggests that individuals who seek out sunlight are also those who spend more time in it. This high sunlight affinity may reinforce a robust circadian rhythm through the ipRGC-SCN pathway [1]. This enhanced circadian entrainment could lead to more consolidated and efficient sleep, potentially allowing for adequate rest within a shorter time frame, thereby reducing the perceived need for longer sleep and decreasing the risk of trouble sleeping.

**In conclusion,** we do not interpret the positive association between sunlight affinity and short sleep as a purely detrimental effect. Instead, we propose that it may represent a lifestyle phenotype where sufficient daytime light exposure promotes circadian health and sleep quality, potentially compensating for a modest reduction in sleep quantity. This hypothesis that sunlight affinity might be linked to more efficient rather than merely deficient sleep needs to be directly tested in future research employing objective measures of both sleep duration and sleep architecture (e.g., actigraphy, polysomnography).

The explanation regarding "short sleep is related to sun exposure" has been revised in the "Discussion" section.

Revised Manuscript with Track Changes (lines 375-382):

“Beyond sunlight exposure behavior, which constitutes one of the two dimensions of sunlight affinity, the subjective propensity for sunlight exposure (SPS) was found to be associated with a concurrent elevation in short sleep and a decline in trouble sleeping when SPS reached a specific threshold (≥4). This suggests that the reduction in sleep duration may not be wholly negative but could be offset by improvements in sleep quality and consolidation, potentially mediated by stronger circadian entrainment. It is plausible that individuals with high sunlight affinity, often engaged in active outdoor lifestyles, may trade some sleep opportunity for these activities yet benefit from the sleep-promoting effects of daytime light exposure. Therefore, this phenomenon suggests that high sunlight affinity may be associated with better sleep quality, but prospective clinical studies are needed to validate this possibility.”

**Reference**

1.	Mahoney HL, Schmidt TM. The cognitive impact of light: illuminating ipRGC circuit mechanisms. Nat Rev Neurosci. 2024;25: 159–175. doi:10.1038/s41583-023-00788-5

***Comment 3: Was there a different between men who worked outdoor jobs vs indoor jobs?***

Response:

We extend our gratitude to the reviewer for highlighting this critical issue. The distinction between outdoor and indoor occupations is indeed a relevant factor that could influence an individual's sunlight exposure patterns and, consequently, the study outcomes. However, the public-use NHANES 2009–2020 files do not contain a variable that directly distinguishes “outdoor” from “indoor” occupations. Therefore, we could not directly stratify participants based on whether they had outdoor or indoor jobs in our analysis.

Nevertheless, our measure of Sunlight Exposure Duration (SED) was designed to capture the behavioral dimension of sunlight affinity by asking participants about the time they spent outdoors (and not under shade) between 9:00 am and 5:00 pm over the past 30 days, including both working and non-working days. This approach aimed to capture each individual's total habitual sunlight exposure, regardless of its source (occupational or recreational).

That said, we acknowledge that occupational sunlight exposure is a significant contributor to total exposure and may have distinct effects. To partially address this, we included Poverty Income Ratio (PIR) as covariates in our multivariate models, which may indirectly capture some socioeconomic aspects related to occupation type. Furthermore, the positive correlation we observed between SPS (psychological preference) and SED (behavioral exposure) suggests that individuals' attitudes align with their behavior, to some extent, irrespective of job type.

We agree with the reviewer that future studies specifically designed to classify occupational sunlight exposure (e.g., using Standard Occupational Classification codes) would provide valuable additional insights. We have added sentences in the Limitations section of the revised manuscript to acknowledge this point. Thank you for this valuable suggestion.

Revised Manuscript with Track Changes (lines 442-445):

“Third, NHANES does not release detailed occupational codes that would allow us to directly classify participants into outdoor versus indoor jobs. Consequently, we could not explore whether the observed associations differ between men with occupational sunlight exposure and those primarily working indoors. Future cycles of NHANES or linked occupational databases could help address this question.”

**Responses to Comments of Reviewer #2:**

***Comment 1: I suspect this is evaluating natural sunlight and not a sun lamp or other artificial sun. You may add a clear statement on this as many who live in northern parts of the country regularly use artificial sun lamps.***

Response:

We are grateful to the reviewer for raising this salient issue. The reviewer is correct that our study specifically evaluated exposure to natural sunlight, not artificial light sources such as sun lamps or light therapy devices. Our measure of Sunlight Exposure Duration (SED) was based solely on participants' self-reported time spent outdoors and not under any shade between 9:00 am and 5:00 pm. This was designed to capture habitual exposure to natural daylight.

We acknowledge that the use of artificial bright light therapy is a common and relevant intervention, particularly in regions with limited natural sunlight. The absence of data on artificial light exposure is a limitation of our study, as it could potentially confound the observed associations.

In response to this comment, we have now added a clear statement in the Discussion section of the manuscript. Thank you for this valuable suggestion, which has helped to improve the clarity and scope of our manuscript.

Revised Manuscript with Track Changes (lines 445-449):

“Fourth, our measure of sunlight exposure (SED) captured only exposure to natural sunlight during daytime hours. We did not have data on exposure to artificial light sources, such as bright light therapy lamps or full-spectrum lighting, which are commonly used, particularly in northern latitudes or for treating seasonal affective disorders. The effects of such artificial light sources on depression and sleep disorders may differ from those of natural sunlight and could represent a potential confounding factor not accounted for in our analysis.”

***Comment 2: OSA is mentioned in the introduction but no part of the modeling for the logistic regression. Other sleep disorders such as OSA, particularly untreated OSA, restless legs, narcolepsy, etc. would have a large impact on the length and quality of sleep.***

Response:

We would like to express our sincere gratitude to the reviewer for this insightful comment. The reviewer's assertion regarding the significance of clinically diagnosed sleep disorders, such as obstructive sleep apnea (OSA), restless legs syndrome, and narcolepsy, as critical confounders is indeed valid. These conditions have the capacity to exert a substantial influence on both sleep duration and quality.

In our analysis, we aimed to adjust for a broad range of health conditions. However, as the reviewer noted, we did not include these specific sleep disorders as covariates in our logistic regression models. This was primarily due to limitations in the NHANES dataset; while it contains data on general "trouble sleeping" and sleep duration, it does not include comprehensive, clinically verified diagnoses for specific sleep disorders like OSA across all survey cycles used in our study.

Despite this limitation, we attempted to indirectly account for the risk of such conditions by adjusting for several known strong correlates and risk factors that are available in NHANES, including:

- Body Mass Index (BMI): A major risk factor for OSA [1].
- Hypertension: A common comorbidity of OSA [2].
- Cardiovascular Disease (CVD): Also linked to OSA [3].

# Diabetes: Another condition associated with sleep disorders [4].

While this approach is undoubtedly imperfect, it was our best available method to partially control for the underlying physiological risks associated with sleep disorders like OSA.

We fully agree with the reviewer that the inability to fully adjust for specific sleep disorders is a limitation of our study, as residual confounding may remain. We have now explicitly acknowledged this important limitation in the revised Discussion section. We strongly advocate that future prospective studies incorporate polysomnography or detailed clinical sleep diagnoses to better disentangle these complex relationships. Thank you for highlighting this crucial point, which significantly strengthens the interpretation of our findings.

Revised Manuscript with Track Changes (lines 449-453):

“Fifth, our models did not adjust for specific clinical sleep disorders such as obstructive sleep apnea, restless legs syndrome, or narcolepsy, which are strong determinants of sleep duration and quality. The NHANES database is deficient in systematic data regarding these clinical diagnoses for all participants. Although we adjusted for key risk factors and comorbidities like BMI, HTN, and CVD, which are proxies for conditions like obstructive sleep apnea, residual confounding remains a possibility.”

# References

1.	Esmaeili N, Gell L, Imler T, Hajipour M, Taranto-Montemurro L, Messineo L, et al. The relationship between obesity and obstructive sleep apnea in four community-based cohorts: an individual participant data meta-analysis of 12,860 adults. eClinicalMedicine. 2025;83: 103221. doi:10.1016/j.eclinm.2025.103221

2.	Brown J, Yazdi F, Jodari-Karimi M, Owen JG, Reisin E. Obstructive Sleep Apnea and Hypertension: Updates to a Critical Relationship. Curr Hypertens Rep. 2022;24: 173–184. doi:10.1007/s11906-022-01181-w

3.	Nguyen D, Hoang C, Truong T, Nguyen D, Lam HG, Sharma A, et al. Multi-level phenotypic models of cardiovascular disease and obstructive sleep apnea comorbidities: A longitudinal Wisconsin sleep cohort study. PLoS One. 2025;20: e0327977. doi:10.1371/journal.pone.0327977

4.	Khalil M, Power N, Graham E, Deschênes SS, Schmitz N. The association between sleep and diabetes outcomes – A systematic review. Diabetes Research and Clinical Practice. 2020;161: 108035. doi:10.1016/j.diabres.2020.108035

***Comment 3: Why was the maximum age 59 years? Older individuals experience high rates of depression in this group and may change the data.***

Response:

We thank the reviewer for this important question regarding the age range of our study population.

The selection of participants aged 20 to 59 years was not an arbitrary choice by our research team, but rather a direct consequence of the NHANES survey design itself. The core variables defining our exposure of interest, Sunlight Preference Score (SPS) and Sunlight Exposure Duration (SED), were only collected by NHANES from adult participants aged 20 to 59 years. These questions were not administered to participants aged 60 years and older in the survey cycles we utilized (2009-2020). Therefore, to ensure the accurate and valid assessment of our primary exposure variable ("sunlight affinity"), we were obliged to restrict our analysis to this age range.

We completely agree with the reviewer that depression and sleep disorders are highly prevalent and clinically significant in older adults. Their exclusion is indeed a limitation, as it restricts the generalizability of our findings to the adult male population aged 60 and above. We had anticipated this concern and had therefore already acknowledged this specific limitation in the Discussion section of our original manuscript.

To further strengthen our manuscript in response to this comment, we have now:

- Added a explicit statement in the Methods section (under “Study population”) clearly explaining the data-driven reason for the age restriction.
- Enhanced the existing paragraph in the Limitations section to more prominently discuss the potential impact of excluding older adults on our results and their generalizability.

We believe these revisions provide crucial context and improve the transparency of our methodology. Thank you for prompting us to make this clearer.

Revised Manuscript with Track Changes (Study population, lines 110-112):

“The NHANES questionnaire modules regarding sunlight preference and exposure duration were administered only to participants aged 20-59 years. Consequently, individuals aged 60 years or older were excluded from this analysis to ensure the valid assessment of the primary exposure variable, sunlight affinity.”

Revised Manuscript with Track Changes (Study population, lines 453-458):

“Sixth, our findings are not generalizable to males aged 60 years and older, as the NHANES protocol for the cycles used did not collect data on sunlight affinity (SPS and SED) in this age group. This is a significant limitation, given the high prevalence of depression and sleep disorders among older adults. The associations between sunlight affinity and mental health outcomes may differ in older populations due to factors such as retirement, altered circadian rhythms, increased chronic disease burden, and reduced mobility. Future studies specifically designed to investigate these relationships in older adult populations are warranted.”

**Responses to Comments of Reviewer #3:**

***Comment 1: Line 97: The reference number of approvals is to be stated.***

Response:

We thank the reviewer for this comment. It is true that the NHANES protocols for each two-year cycle (2009–2020) received separate ethical approval, each with its own unique approval number (Table: Approved NHANES protocols). Rather than listing each individual approval number, which may hinder readability, we have provided a direct link to the official NHANES ERB (Ethics Review Board) website where all approval references are publicly accessible:
[https://www.cdc.gov/nchs/nhanes/about/erb.html](https://www.cdc.gov/nchs/nhanes/about/erb.html)

This allows interested readers to easily access the complete and detailed approval information for each cycle. We appreciate the reviewer’s attention to this important methodological detail.

**Table: Approved NHANES protocols**

| Survey Name and Date | NCHS ERB Protocol Number or Description  (<https://www.cdc.gov/nchs/nhanes/about/erb.html>) |
| --- | --- |
| NHANES 2019-2020 | Continuation of Protocol #2018-01 |
| NHANES 2017-2018 | Protocol #2018-01 Effective beginning October 26, 2017 Continuation of Protocol #2011-17 Effective through October 26, 2017 |
| NHANES 2015-2016 | Continuation of Protocol #2011-17 |
| NHANES 2013-2014 | Continuation of Protocol #2011-17 |
| NHANES 2011-2012 | Protocol #2011-17 |
| NHANES 2009-2010 | Continuation of Protocol #2005-06 |

NCHS, National Center for Health Statistics; ERB, Ethics Review Board.

***Comment 2: Line 145-149: It is unclear how the sleep hours were derived. More information is to be provided.***

Response:

We appreciate the reviewer's feedback, as it has enabled us to offer a more thorough explanation of our approach. The derivation of "sleep hours" was based on self-reported data from the NHANES questionnaire. However, the specific survey items used changed between the 2009–2014 and 2015–2020 cycles:

- **For NHANES cycles 2009–2014, sleep hours were based on the direct response to the question SLD010H: "How much sleep do you usually get at night on weekdays or workdays?" (Answer in hours).**
- **For NHANES cycles 2015–2020,** the direct question was replaced. Sleep hours were calculated from two separate items:

**SLQ300: "**What time do you usually go to sleep on weekdays or workdays?**"**

**SLQ310: "**What time do you usually wake up on weekdays or workdays?**"**

We computed the sleep duration by calculating the time difference between the reported bedtime and wake time. This calculated value (in hours) serves as the equivalent measure for the earlier cycles' direct question.

Despite the change in data collection, the resulting variable in both cases is a continuous measure of **usual nightly sleep duration on weekdays (in hours)**.

We apologize for the lack of clarity in the original manuscript. We have now revised the **Methods** section ("Assessment of depression and sleep disorders") to include these specific NHANES item numbers and a clearer explanation of the derivation process, as detailed below. Thank you for prompting this important clarification.

Revised Manuscript with Track Changes (lines 147-153):

“Sleep hours were derived from self-reported sleep time. For participants in the 2009–2014 survey cycles, this was based on the direct question "How much sleep do you usually get at night on weekdays or workdays?" (NHANES variable SLD010H; answer in hours). For the 2015–2020 cycles, sleep duration was calculated as the time difference between the responses to " What time do you usually go to sleep on weekdays or workdays?" (SLQ300) and "What time do you usually wake up on weekdays or workdays?" (SLQ310) [25]. This computed value (in hours) provided a consistent measure of usual weekday sleep duration across all cycles. Individuals reporting sleep hours of fewer than 7 were categorized as having short sleep [26].”

# References

25.	Chen R, Han J, Li S, Deng H, Jian T, Huang Z, et al. Interaction between sleep duration and physical activity on mortality among cancer survivors: findings from National Health and Nutrition Examination Surveys 2007–2018. Front Public Health. 2025;13: 1532320. doi:10.3389/fpubh.2025.1532320

26.	Chaput J-P, McHill AW, Cox RC, Broussard JL, Dutil C, Da Costa BGG, et al. The role of insufficient sleep and circadian misalignment in obesity. Nat Rev Endocrinol. 2023;19: 82–97. doi:10.1038/s41574-022-00747-7

***Comment 3: Line 167: Q1, Q3 is to be replaced with IQR.***

Response:

We sincerely appreciate the reviewer's meticulous attention to statistical terminology. This is a valuable suggestion to enhance the clarity and standardization of our data presentation.

As recommended, we have revised the description in the Statistical methodology section (Line 167). The original phrase:
"data were expressed as median (Q₁, Q₃)"
has been replaced with:
"data were expressed as median (IQR, interquartile range)"

This change has been implemented throughout the manuscript to ensure consistent use of standard statistical notation. Thank you for this helpful correction.

Revised Manuscript with Track Changes (lines 147-153):

**Table 1. Characteristics of participants by depression and sleep disorders in baseline.**

| **Variable** | **Non-depression**  **(n = 5909)** | **StD**  **(n = 1008)** | **MDD**  **(n = 389)** | **P** | **Non-short sleep**  **(n = 4604)** | **Short sleep**  **(n = 2702)** | **P** | **Non-trouble sleeping**  **(n = 5820)** | **Trouble sleeping**  **(n = 1486)** | **P** |
| --- | --- | --- | --- | --- | --- | --- | --- | --- | --- | --- |
| **Age (year), M (IQR)** | 39.00 (20.00) | 38.00 (20.00) | 38.00 (21.00) | 0.659 | 38.00 (21.00) | 40.00 (18.00) | <0.001 | 38.00 (20.00) | 43.00 (19.00) | <0.001 |
| **PIR, M (IQR)** | 3.43 (3.25) | 2.65 (3.32) | 1.85 (3.17) | <0.001 | 3.31 (3.36) | 3.19 (3.37) | 0.150 | 3.14 (3.40) | 3.73 (3.22) | <0.001 |
| **BMI (kg/m^2^), M (IQR)** | 28.00 (7.25) | 28.30 (7.6) | 28.00 (8.70) | 0.393 | 27.87 (7.30) | 28.50 (7.40) | 0.003 | 27.80 (7.20) | 29.20 (8.20) | <0.001 |

StD, subthreshold depression; MDD, major depressive disorder; SE, standard error; BMI, body mass index; CVD, cardiovascular disease; SPS, sunlight preference score; SED, sunlight exposure duration; M, median; IQR, interquartile range.

***Comment 4: OR to be replaced with aOR, where possible in the text/tables, to differentiate crude and adjusted.***

Response:

We sincerely thank the reviewer for this excellent suggestion. We have now replaced "OR" with "aOR" (adjusted Odds Ratio) throughout the entire text as well as in all tables and figures wherever the estimates come from the adjusted models. This change significantly enhances the clarity and precision of our results by explicitly differentiating between crude and adjusted estimates, thereby preventing any potential confusion for the reader. We appreciate this valuable input, which strengthens the methodological rigor of our manuscript.

***Comment 5: The fulfilment of statistical tests assumptions is to be stated e,g, multicollinearity etc.***

Response:

We are grateful to the reviewer for raising this crucial point regarding the verification of statistical assumptions. Ensuring the robustness of our multivariate models is paramount.

To assess multicollinearity in our fully adjusted logistic regression models (Model 3), we calculated the variance inflation factors (VIF) for all included covariates. As shown in the table below, all VIF values were substantially below the common threshold of 5 (and indeed below 3), confirming that severe multicollinearity was not present and that the model estimates are stable and reliable.

****Table: Variance inflation factors (VIF) for covariates in the fully adjusted model.****

| **Covariates** | **VIF** | **Covariates** | **VIF** |
| --- | --- | --- | --- |
| Age | 1.290 | Physical activity | 1.067 |
| Race | 1.362 | Cardiovascular disease | 1.070 |
| Education | 1.380 | Liver condition | 1.032 |
| Marital status | 1.251 | Asthma | 1.024 |
| PIR | 1.259 | Diabetes | 1.177 |
| BMI | 1.175 | Hypertension | 1.198 |
| Smoking status | 1.041 | Weak/failing kidneys | 1.029 |
| Alcohol use | 1.119 | Cancer/Malignancy | 1.025 |

PIR, poverty income ratio; BMI, body mass index.

In accordance with this comment, we have added the following statement to the Statistical methodology section (Lines 182-183) of the revised manuscript: "The absence of substantial multicollinearity was confirmed in the fully adjusted model (Model 3), with all variance inflation factors being below 3."

We extend our appreciation to the reviewer for this suggestion, which has undoubtedly strengthened the methodological transparency of our work.

***Comment 6: Model fit for each statistical test, where applicable, is to be presented.***

Response:

We sincerely thank the reviewer for this critical suggestion, which has significantly strengthened the methodological rigor of our manuscript. In full accordance with your comment, we have now comprehensively evaluated and presented the goodness-of-fit for our statistical models. **Specifically, we have taken the following actions:**

- **Model fit assessment added to Methods:** We have revised the **Statistical methodology** section to explicitly state that model fit was assessed using the Akaike information criterion (AIC) and the Bayesian information criterion (BIC) for the multivariable logistic regression models.
- **Detailed results presented in manuscript:** As requested, the results of the model fit assessment are now presented **directly within the Results section**. We have added a new subsection and a dedicated S4 Table (please see revised manuscript) that reports the AIC and BIC values for the crude (Model 1), demographically-adjusted (Model 2), and fully-adjusted (Model 3) models for each of the four primary outcomes (StD, MDD, short sleep, and trouble sleeping).
- **Key finding on model improvem**ent: The results demonstrate a substantial and monotonic decrease in both AIC and BIC values from Model 1 to Model 3 for all outcomes. This provides strong, quantitative evidence that the sequential addition of covariate sets (demographics, followed by lifestyle and comorbidities) consistently improved the fit of our models to the data without overfitting.

We believe that the presentation of these criteria directly addresses your comment and provides readers with a transparent assessment of the robustness of our primary models. The addition of this analysis undoubtedly enhances the quality and credibility of our work, and we are grateful for your guidance.

Revised Manuscript with Track Changes (lines 246-249):

“The overall fit of the fully adjusted logistic regression models (Model 3) was assessed. The AIC and BIC values for each model are presented in Table 3. Lower values indicate superior model fit. For all outcome variables, the AIC and BIC decreased substantially from the crude (Model 1) to the fully adjusted models (Model 3), demonstrating that the inclusion of demographic, lifestyle, and comorbidity covariates significantly improved model fit (S4 Table).”

Revised Manuscript with Track Changes (S4 Table):

**S4 Table. Goodness-of-fit statistics for the logistic regression models.**

| **Variables** | **Model** | **StD** | | **MDD** | | **Short sleep** | | **Trouble sleeping** | |
| --- | --- | --- | --- | --- | --- | --- | --- | --- | --- |
|  |  | **AIC** | **BIC** | **AIC** | **BIC** | **AIC** | **BIC** | **AIC** | **BIC** |
| **SPS (scores)** | Model 1 | 5737 | 5763 | 2917 | 2931 | 9628 | 9655 | 7375 | 7389 |
|  | Model 2 | 5626 | 5750 | 2742 | 2830 | 9524 | 9641 | 7104 | 7193 |
|  | Model 3 | 5592 | 5715 | 2655 | 2823 | 9482 | 9614 | 6825 | 6997 |
| **SED (hours)** | Model 1 | 5748 | 5773 | 2918 | 2931 | 9626 | 9660 | 7373 | 7387 |
|  | Model 2 | 5639 | 5762 | 2733 | 2821 | 9528 | 9640 | 7108 | 7197 |
|  | Model 3 | 5602 | 5728 | 2645 | 2814 | 9488 | 9618 | 6824 | 6997 |

SPS, sunlight preference score; SED, sunlight exposure duration; StD, subthreshold depression; MDD, major depressive disorder; AIC, Akaike information criterion; BIC, Bayesian information criterion.

Model 1 was without adjustment for covariates. Model 2 adjusted for demographic covariates (including age, race, education, marital status and PIR). Model 3 further adjusted for lifestyle (including smoking history, alcohol consumption history, physical activity, and BMI) and comorbidities (including CVD, liver condition, asthma, weak/failing kidneys, cancer/malignancy, diabetes, and hypertension) based on Model 2.

***Comment 7: Line 260: 20–40 and 40–60 years categories seem overlap.***

Response:

We truly appreciate the reviewers' meticulous attention to detail. This is indeed a valuable correction. We apologize for the oversight in the initial description of the age group categorization (20–40 and 40–60 years), which created an overlap at the 40-year boundary. We confirm that this was merely a typographical error in the manuscript, and that all actual statistical computations were correctly performed using the non-overlapping groups of 20–39 years and 40–59 years.

In response to this comment, we have carefully revised the manuscript. The age groups for the subgroup analysis have been corrected to 20–39 years and 40–59 years throughout the text and in all relevant figures (e.g., Fig 3, Fig 4) to ensure clear and non-overlapping intervals. Thank you for this helpful suggestion, which has improved the clarity and precision of our work.

Revised Manuscript with Track Changes (Figs 3 and 4):

**Fig 3. Subgroup analyses of the associations between sunlight preference score and depression and sleep disorders.**

**Fig 4. Subgroup analyses of the associations between sunlight exposure duration and depression and sleep disorders.**

***Comment 8: Figure 2: Legend to be provided and more detailed information is to be provided, e.g., model details, statistical test for p non-linear, etc.***

Response:

The author expresses their gratitude to the referee for this valuable suggestion. We completely agree that the figure legend should be comprehensive and self-explanatory. We have now revised the legend for Figure 2 to include the following key details:

- A clear description of what the solid lines and shaded areas represent.
- The Definition of reference points among models.
- The exact list of all covariates included in the fully adjusted model (Model 3).
- The explicit p-value for the nonlinear relationship in each individual subplot (A–H).

We believe the revised legend now provides all necessary information for the reader to interpret the figure accurately without referring to the main text. Thank you for helping us improve the clarity of our presentation.

Revised Manuscript with Track Changes (lines 250-267):

**Fig 2. Restricted cubic spline analyses of the relationships between sunlight affinity and depression and sleep disorders.**

SPS, sunlight preference score; SED, sunlight exposure duration; StD, subthreshold depression; MDD, major depressive disorder; aOR, adjusted odds ratio; CI, confidence interval.

(A) SPS is associated with StD, but no significant nonlinear relationship is observed (P for overall = 0.002; P for non-linear = 0.263).

(B) SPS is associated with MDD, but no significant nonlinear relationship is observed (P for overall < 0.001; P for non-linear = 0.062).

(C) SED demonstrates a significant nonlinear association with StD (P for overall = 0.006; P for non-linear = 0.018).

(D) SED is associated with MDD, but no significant nonlinear relationship is observed (P for overall = 0.001; P for non-linear = 0.088).

(E) SPS shows a significant nonlinear association with short sleep (P for overall < 0.001; P for non-linear = 0.003).

(F) SPS is associated with trouble sleeping, but no significant nonlinear relationship is observed (P for overall = 0.006; P for non-linear = 0.074).

(G) SED shows a significant nonlinear association with short sleep (P for overall < 0.001; P for non-linear = 0.001).

(H) SED demonstrates a significant nonlinear association with trouble sleeping (P for overall < 0.001; P for non-linear = 0.018).

Solid lines represent the aORs, and shaded areas represent the 95% CIs. The reference values were set at the median value of the SPS or SED. All models were adjusted for demographic covariates (including age, race, education, marital status and PIR), lifestyle (including smoking history, alcohol consumption history, physical activity, and BMI) and comorbidities (including CVD, liver condition, asthma, weak/failing kidneys, cancer/malignancy, diabetes, and hypertension).

***Comment 9: Line 178: Likelihood tests to be replaced with likelihood ratio tests. Likewise with S6Table.***

Response:

We are grateful to the reviewer for this precise terminology correction. We have revised the text on Line 178 of the manuscript and S6 Table, replacing "likelihood tests" with the statistically precise term "likelihood ratio tests". This change enhances the methodological accuracy of our description. We truly appreciate the reviewer's expertise, which has helped us improve the clarity and professionalism of our work.

***Comment 10: Line 184 DE; symbol ; is to be replaced with semi-colon:***

Response:

We thank the reviewer for their sharp eye for detail in catching this punctuation error. We have revised the sentence on Line 184 accordingly, replacing the semicolon after DE with a colon to properly introduce the definition of the term. We appreciate this careful correction, which improves the grammatical precision of our manuscript.

Revised Manuscript with Track Changes (lines 192-193):

“Total effect (TE) was derived as the sum of IE and direct effect (DE: exposure–outcome association adjusted for the mediator).”

***Comment 11: Line 189: Model fit to be mentioned and presented in the results section.***

Response:

We are truly grateful to the reviewer for emphasizing the importance of transparent model reporting. This suggestion has significantly enhanced the methodological rigor of our manuscript. In direct response, we have not only added a description of the model fit assessment (using AIC and BIC) in the Statistical methodology section but have also dedicated a new subsection within the Results to present the fit statistics for our primary models. Furthermore, we have created a new S4 Table to comprehensively display the AIC and BIC values for all nested models (Model 1, 2, and 3) across the four main outcomes. This valuable comment ensures our readers can fully evaluate the robustness of our analytical models.

Revised Manuscript with Track Changes (Statistical methodology, lines 198???-200):

“The goodness-of-fit for multivariable logistic regression models was assessed using the Akaike information criterion (AIC) and the Bayesian information criterion (BIC), with lower values indicating a better balance of model fit and parsimony.”

Revised Manuscript with Track Changes (lines 246-249):

“The overall fit of the fully adjusted logistic regression models (Model 3) was assessed. The AIC and BIC values for each model are presented in Table 3. Lower values indicate superior model fit. For all outcome variables, the AIC and BIC decreased substantially from the crude (Model 1) to the fully adjusted models (Model 3), demonstrating that the inclusion of demographic, lifestyle, and comorbidity covariates significantly improved model fit (S4 Table).”

Revised Manuscript with Track Changes (S4 Table):

**S4 Table. Goodness-of-fit statistics for the logistic regression models.**

| **Variables** | **Model** | **StD** | | **MDD** | | **Short sleep** | | **Trouble sleeping** | |
| --- | --- | --- | --- | --- | --- | --- | --- | --- | --- |
|  |  | **AIC** | **BIC** | **AIC** | **BIC** | **AIC** | **BIC** | **AIC** | **BIC** |
| **SPS (scores)** | Model 1 | 5737 | 5763 | 2917 | 2931 | 9628 | 9655 | 7375 | 7389 |
|  | Model 2 | 5626 | 5750 | 2742 | 2830 | 9524 | 9641 | 7104 | 7193 |
|  | Model 3 | 5592 | 5715 | 2655 | 2823 | 9482 | 9614 | 6825 | 6997 |
| **SED (hours)** | Model 1 | 5748 | 5773 | 2918 | 2931 | 9626 | 9660 | 7373 | 7387 |
|  | Model 2 | 5639 | 5762 | 2733 | 2821 | 9528 | 9640 | 7108 | 7197 |
|  | Model 3 | 5602 | 5728 | 2645 | 2814 | 9488 | 9618 | 6824 | 6997 |

SPS, sunlight preference score; SED, sunlight exposure duration; StD, subthreshold depression; MDD, major depressive disorder; AIC, Akaike information criterion; BIC, Bayesian information criterion.

Model 1 was without adjustment for covariates. Model 2 adjusted for demographic covariates (including age, race, education, marital status and PIR). Model 3 further adjusted for lifestyle (including smoking history, alcohol consumption history, physical activity, and BMI) and comorbidities (including CVD, liver condition, asthma, weak/failing kidneys, cancer/malignancy, diabetes, and hypertension) based on Model 2.

***Comment 12: S3 Table: typo withdepression***

Response:

We sincerely thank the reviewer for their meticulous review and for identifying this typographical error. We apologize for the oversight and have corrected "withdepression" to "with depression" in S3 Table. We greatly appreciate the reviewer's attention to detail, which has helped us improve the accuracy and professionalism of our manuscript.

***Comment 13: S4 Table: The decimal points are to be standardized.***

Response:

We extend our sincere thanks to the reviewer for this precise feedback, which ensures consistency in our data presentation. We have standardized the decimal places for all P-values to three digits after the decimal point and β (95% CI) to four digits after the decimal point in S4 Table (now is S5 Table). We greatly value the reviewer's diligence, as this refinement enhances the readability and professionalism of our supplementary materials.

Revised Manuscript with Track Changes (S5 Table):

**S5 Table. Mediation analyses results.**

| **Exposure → Mediator → Outcome** | **Effect Type** | **β (95% CI)** | **P** | **Prop. Mediated (95% CI)** |
| --- | --- | --- | --- | --- |
| Sleep hours → SPS → PHQ-9 scores | Indirect effect | 0.0126 (0.0043, 0.0200) | <0.001 | -0.300 (-0.389, -0.030) |
|  | Direct effect | -0.0544 (-0.2250, -0.0300) | 0.004 |  |
|  | Total Effect | -0.0419 (-0.2150, -0.0300) | 0.014 |  |
| PHQ-9 scores → SPS → Sleep hours | Indirect effect | 0.0015 (0.0006, 0.0020) | <0.001 | -0.290 (-0.390, -0.020) |
|  | Direct effect | -0.0068 (-0.0320, 0.0000) | 0.004 |  |
|  | Total Effect | -0.0053 (-0.0300, 0.0000) | 0.014 |  |
| Trouble sleeping → SPS → PHQ-9 scores | Indirect effect | 0.0069 (0.0015, 0.0300) | 0.020 | 0.003 (0.001, 0.010) |
|  | Direct effect | 2.4917 (2.2310, 2.8000) | <0.001 |  |
|  | Total Effect | 2.4986 (2.2490, 2.8200) | <0.001 |  |
| PHQ-9 scores → SPS → Trouble sleeping | Indirect effect | 0.0000 (-0.0001, 0.0000) | 0.240 | 0.001 (-0.002, 0.010) |
|  | Direct effect | 0.0279 (0.0230, 0.0300) | <0.001 |  |
|  | Total Effect | 0.0279 (0.0230, 0.0300) | <0.001 |  |
| StD → SPS → Short Sleep | Indirect effect | -0.0026 (-0.0060, 0.0000) | <0.001 | -0.039 (-0.073, -0.010) |
|  | Direct effect | 0.0698 (0.0660, 0.1400) | <0.001 |  |
|  | Total Effect | 0.0672 (0.0620, 0.1400) | <0.001 |  |
| Short Sleep → SPS → StD | Indirect effect | -0.0016 (-0.0032, 0.0000) | <0.001 | -0.042 (-0.073, -0.010) |
|  | Direct effect | 0.0387 (0.0350, 0.0800) | <0.001 |  |
|  | Total Effect | 0.0371 (0.0330, 0.0800) | <0.001 |  |
| MDD → SPS → Trouble sleeping | Indirect effect | 0.0019 (-0.0006, 0.0019) | 0.130 | 0.004 (-0.001, 0.020) |
|  | Direct effect | 0.5228 (0.3770, 0.5000) | <0.001 |  |
|  | Total Effect | 0.5247 (0.3800, 0.5000) | <0.001 |  |
| Trouble sleeping → SPS → MDD | Indirect effect | 0.0020 (0.0001, 0.0020) | 0.022 | 0.011 (0.001, 0.020) |
|  | Direct effect | 0.1769 (0.1210, 0.1800) | <0.001 |  |
|  | Total Effect | 0.1789 (0.1220, 0.1800) | <0.001 |  |
| PHQ-9 scores → SED → Sleep hours | Indirect effect | 0.0016 (0.0004, 0.0020) | <0.001 | -0.305 (-0.303, -0.020) |
|  | Direct effect | -0.0069 (-0.0320, 0.0000) | 0.004 |  |
|  | Total Effect | -0.0053 (-0.0300, 0.0000) | 0.014 |  |
| Sleep hours → SED → PHQ-9 scores | Indirect effect | 0.0129 (0.0025, 0.0100) | <0.001 | -0.309 (-0.300, -0.010) |
|  | Direct effect | -0.0548 (-0.2220, -0.0300) | 0.004 |  |
|  | Total Effect | -0.0419 (-0.2150, -0.0300) | 0.014 |  |
| PHQ-9 scores → SED → Trouble sleeping | Indirect effect | 0.0003 (-0.0000, 0.0000) | 0.060 | 0.010 (-0.000, 0.010) |
|  | Direct effect | 0.0276 (0.0230, 0.0300) | <0.001 |  |
|  | Total Effect | 0.0279 (0.0230, 0.0300) | <0.001 |  |
| Trouble sleeping → SED → PHQ-9 scores | Indirect effect | 0.0194 (0.0059, 0.0300) | 0.002 | 0.008 (0.002, 0.010) |
|  | Direct effect | 2.4793 (2.2320, 2.8000) | <0.001 |  |
|  | Total Effect | 2.4986 (2.2490, 2.8200) | <0.001 |  |
| Short Sleep → SED → Trouble sleeping | Indirect effect | -0.0045 (-0.0050, 0.0000) | 0.002 | -0.132 (-0.074, -0.010) |
|  | Direct effect | 0.0386 (0.0350, 0.0900) | <0.001 |  |
|  | Total Effect | 0.0341 (0.0330, 0.0800) | <0.001 |  |
| Trouble sleeping → SED → Short Sleep | Indirect effect | -0.0055 (-0.0050, 0.0000) | 0.002 | -0.129 (-0.071, -0.010) |
|  | Direct effect | 0.0482 (0.0440, 0.1100) | <0.001 |  |
|  | Total Effect | 0.0427 (0.0410, 0.1100) | <0.001 |  |

***Comment 14: Some references do not conform to the journal’s required format***

Response:

We sincerely appreciate the reviewer's diligence in highlighting the inconsistencies in our reference formatting. We have conducted a thorough, line-by-line review of all references and have meticulously reformatted them to ensure full compliance with the journal's required style. This attention to detail significantly improves the professionalism and uniformity of our manuscript, and we are very grateful for the guidance.

Revised Manuscript with Track Changes (lines 523-530):

1.	Louise Adams, Laura Marshall. Comorbid depression in medical diseases. Nat Rev Dis Primers. 2020;6: 70. doi:10.1038/s41572-020-0211-z

2.	Hu C, Ding L, Peng K. Global burden of major depressive disorders attributable to intimate partner violence against women: Magnitude, temporal trends, and regional inequalities. Journal of Affective Disorders. 2024;363: 182–191. doi:10.1016/j.jad.2024.07.098

3.	Nathan Swetlitz. Depression’s Problem With Men. AMA Journal of Ethics. 2021;23: E586-589. doi:10.1001/amajethics.2021.586

We are truly grateful to the editorial team and reviewer for their thorough evaluation and insightful feedback, which have significantly enhanced our manuscript. The constructive feedback has been incredibly beneficial, enhancing the scientific rigor of our work and expanding its translational relevance. We are extremely grateful for the time, expertise, and thoughtful engagement demonstrated throughout this review process. The revised manuscript now addresses all the concerns raised and demonstrates significantly enhanced scientific rigor. We believe that the current version meets the journal's publication standards and hope that this manuscript will be accepted and published.

Thank you very much for your attention and time. We look forward to hearing from you.

Yours sincerely,

Jia Yang

E-mail: yangjia202416@163.com

September 21th, 2025

College of Chinese Medicine, Changchun University of Chinese Medicine, Changchun City, Jilin Province, China
